# Asporin enhances colorectal cancer metastasis through activating the EGFR/Src/cortactin signaling pathway

**DOI:** 10.18632/oncotarget.12336

**Published:** 2016-09-29

**Authors:** Huo Wu, Xiaoqian Jing, Xi Cheng, Yonggang He, Lei Hu, Haoxuan Wu, Feng Ye, Ren Zhao

**Affiliations:** ^1^ Department of General Surgery, Shanghai Institute of Digestive Surgery, Ruijin Hospital, Shanghai Jiaotong University School of Medicine, Shanghai, China

**Keywords:** asporin, colorectal cancer, cortactin, metastasis

## Abstract

Asporin has been implicated as an oncogene in various types of human cancers; however, the roles of asporin in the development and progression of colorectal cancer (CRC) have not yet been determined. With clinical samples, we found that asporin was highly expressed in CRC tissues compared to adjacent normal tissues and the asporin expression levels were significantly associated with lymph node metastasis status and TNM stage of the patients. Through knockdown of asporin in CRC cell lines RKO and SW620 or overexpression of asporin in cell lines HT-29 and LoVo, we found that asporin could enhance wound healing, migration and invasion abilities of the CRC cells. Further more, with the human umbilical vein endothelial cells (HUVECs) tube formation assays and the xenograft model, we found that asporin promoted the tumor growth through stimulating the VEGF signaling pathway. The portal vein injection models suggested that asporin overexpression stimulated the liver metastasis of HT29 cell line, while asporin knockdown inhibited the liver metastasis of RKO cell line. In addition, asporin was found to augment the phosphorylation of EGFR/Src/cortactin signaling pathway, which might be contributed to the biological functions of asporin in CRC metastasis. These results suggested that asporin promoted the tumor growth and metastasis of CRC, and it could be a potential therapeutic target for CRC patients in future.

## INTRODUCTION

Colorectal cancer (CRC) is one of the most common causes of cancer-related death worldwide [1]. With increased adoption of western lifestyles, high CRC rates have been reported in developing countries in which the risk was once low [2]. Early stage CRC symptoms are often minimal, resulting in diagnostic delay and dismal survival for late stage patients. Patients with metastatic CRC have a 5-year survival rate of only 11.7% [3]. Elucidating the molecular mechanisms involved in the initiation and progression of CRC may provide biomarkers for early detection and targeted therapy.

Asporin, also known as periodontal ligament-associated protein1 (PLAP1), is an extracellular matrix (ECM) protein. It belongs to the small leucine-rich proteoglycans (SLRPs) family and contains a unique aspartate-rich N terminus that distinguishes it from other SLRPs family members [4]. It was firstly identified in human cartilage [5] and it was associated with the pathogenesis of bone and joint diseases, including osteoarthritis [6], intervertebral disc degeneration [7] and periodontal ligament mineralization [8]. Recently, asporin was found to play important roles in gastric cancer [9, 10], pancreatic cancer [11, 12] and prostate cancer [13]. However, whether asporin is involved in the CRC development and progression has not been clarified. Here, for the first time, we evaluated the clinical relevance of the asporin expression in CRC and determined the underlying mechanisms for the oncogenic activities of asporin in the CRC development and progression.

## RESULTS

### Asporin is overexpressed in CRC tissues

**Figure 1:**
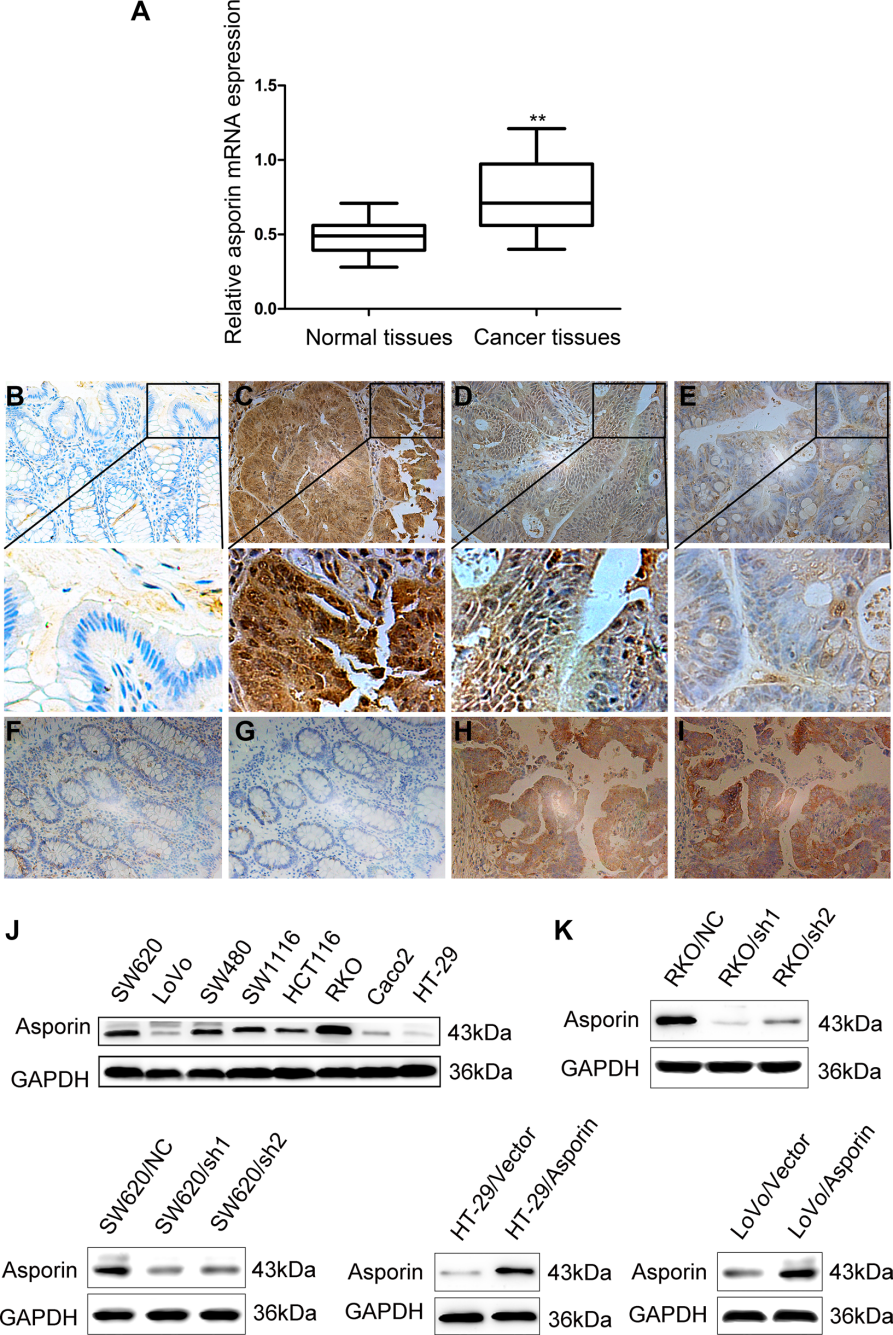
Expression of asporin in the CRC tissues and cell lines (200×) (**A**) Asporin mRNA expression in CRC tissues and paired adjacent non-tumor tissues were analyzed by qRT-PCR. Data were presented as 2^−ΔΔCt^. (**B**) Negative asporin expression in adjacent normal mucosa examined with immunohistochemical staining. (**C**–**E)**. Immunohistochemical results of asporin expression in CRC tissues which were classified as strong positive (C), weak positive (D) and negative (E). (**F**–**G**). Negative expression of asporin and p-cortactin (Tyr421) in adjacent normal mucosa. (**H**–**I)**. Co-expression of asporin and p-cortactin (Tyr421) in human CRC tissues. (**J**) Asporin protein expression in CRC cells analyzed by western blotting methods. (**K**) Suppression and overexpression of asporin in CRC cells were confirmed by western blotting. GAPDH was used as a loading control. ^**^p < 0.01.

We firstly assessed the expression of asporin in colorectal tissues by qRT-PCR and immunohistochemistry staining methods. The mRNA level of asporin in CRC tissues was significantly higher compared to the adjacent non-tumor tissues (*p* < 0.001, Figure 1A). Immunohistochemical staining revealed significantly increased asporin staining (136/200) in the CRC tissues compared to the matching non-tumor tissues (Figure 1B, 1C, 1D and 1E). The clinicopathological features of the 200 included patients were summarized in Table 1 and statistical analyses suggested that asporin expression levels were significantly correlated with lymph node metastasis status and TNM stage of the patients. No significant relationships between asporin expression and other clinicopathologic features, including gender, age, and tumor size were found (Table 1).

**Table 1:** Associations between asporin expression and clinicopathologic variables in 200 CRC patients

Variables	Number of cases	Asporin immunostaining		*P*-value
		Positive (*n* = 136)	Negative (*n* = 64)	
Gender				
male	126	87	39	0.679
female	74	49	25	
Age(years)				
≥ 65	80	52	28	0.458
< 65	120	84	36	
Tumor size(cm)				
≥ 5	71	50	21	0.586
< 5	129	86	43	
T stage				
T1, T2	84	53	31	0.206
T3, T4	116	83	33	
Lymph node metastasis				
Negative	113	70	43	0.036
Positive	87	66	21	
Positive lymph node				
N1	64	45	19	0.044
N2	23	21	2	
Distant metastasis				
Negative	187	125	62	0.184
Positive	13	11	2	
Differentiation				
Undifferentiated, poorly	65	40	25	0.174
Moderated, well	135	96	39	
TNM stage				
I+II	113	67	46	0.003
III+IV	87	69	18	

### Construction of stable asporin knockdown and overexpression CRC cell lines

We explored the asporin expression in a series of CRC cell lines, including HT-29, LoVo, Caco2, HCT116, SW1116, SW480, RKO and SW620. Among these cell lines, RKO and SW620 exhibited relatively higher levels of asporin expression while HT-29 and LoVo showed lower levels of asporin (Figure 1J). Immunohistochemistry staining of CRC cells showed that asporin was located in the cytoplasm of cells. The immunohistochemitry staining results were similar with the western blotting results (Supplementary Figure S1). To further explore the functions of asporin in CRC cell lines, we applied shRNA to knockdown asporin expression in RKO and SW620 cell lines (Figure 1K). Additionally, we overexpressed asporin expression in cell lines HT-29 and LoVo using the lentivirus system (Figure 1K). We also determined the levels of asporin in the conditional medium of asporin knockdown or overexpressed cell lines and found they were consistent with the results in the whole cell levels (Supplementary Figure S2).

### Asporin promotes wound healing, migration and invasion of CRC cells

**Figure 2:**
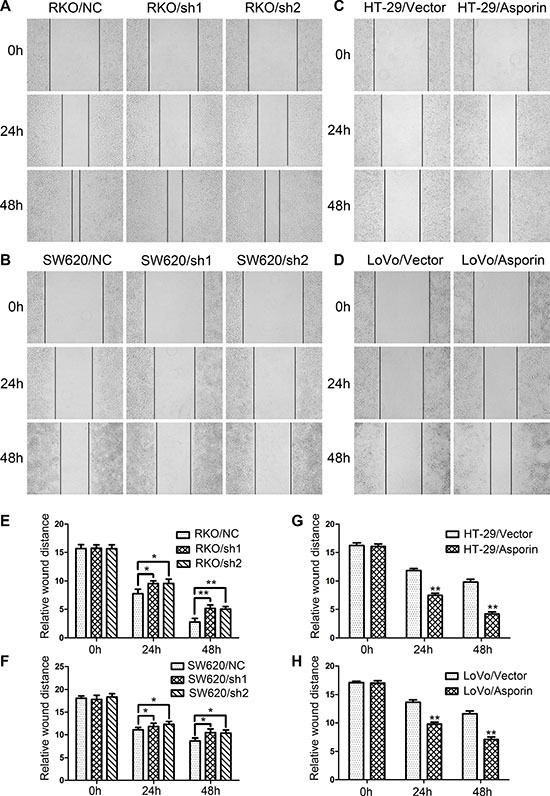
Asporin promotes wound healing of CRC cells (**A**–**D**) Representative photographs of wound healing assays. Photographs were taken 0, 24 and 48 hours after scratching (200×). (**E**–**H)**. Relative distances of wound edges of CRC cells. Data are represented as mean ± SD of three independent experiments. ^*^p < 0.05; ^**^p < 0.01.

To get insight into the potential roles of asporin as an oncogene that might influence the invasion of the CRC cells, we determined the wound healing abilities of CRC cells with asporin knockdown. After 48 hours incubation, the size of the residual scratch wound in RKO/sh1-asporin, RKO/sh2-asporin and SW620/sh1-asporin, SW620/sh2-asporin cells were significantly larger than their corresponding control cells (Figure 2A, 2B, 2E and 2F). In contrast, a shorter distance was noticed in asporin overexpression cells HT-29 and LoVo when compared with the control cells (Figure 2C, 2D, 2G and 2H).

**Figure 3:**
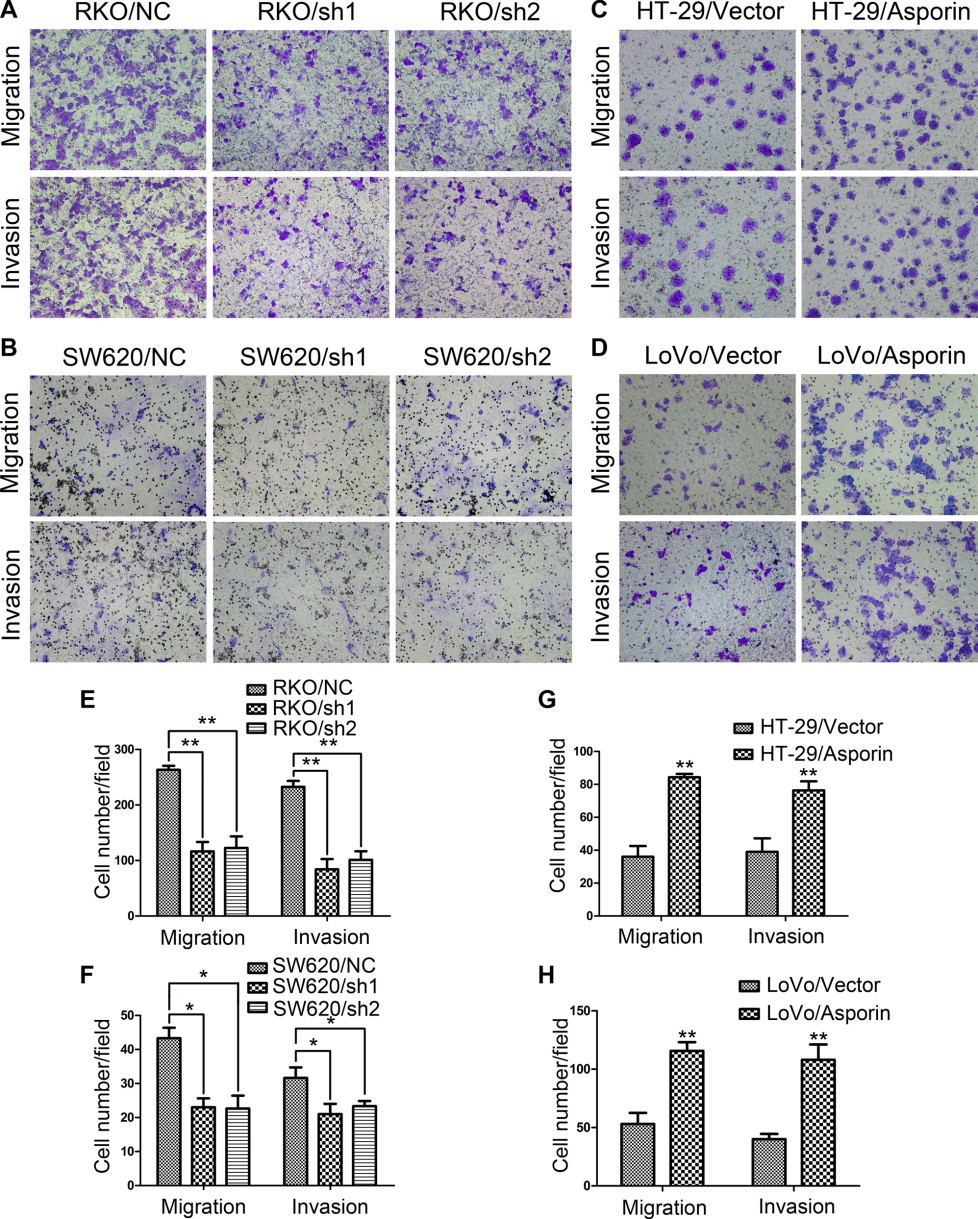
Asporin enhances migration and invasion of CRC cells (**A**–**D**) Representative photographs of migratory and invasive cells on the membrane in transwell assays (200×). (**E**–**H)**. Average numbers of migrated cells and invaded cells. Data are represented as mean ± SD of three independent experiments. ^*^p < 0.05; ^**^p < 0.01.

Transwell assays were performed to further evaluate the influences of asporin on cellular migration and invasion abilities. After 24 hours incubation, cells were counted under an inverted microscope. The number of cells that migrated into the lower chamber was significantly less in RKO/sh1-asporin, RKO/sh2-asporin, and SW620/sh1-asporin, SW620/sh2-asporin cells compared to their corresponding control cells both in the migration and invasion assays (Figure 3A, 3B, 3E and 3F). In contrast, overexpression of asporin clearly augmented cell migration and invasion abilities of both HT-29 and LoVo cells (Figure 3C, 3D, 3G and 3H).

### Overexpression of asporin stimulates endothelial tube formation

**Figure 4:**
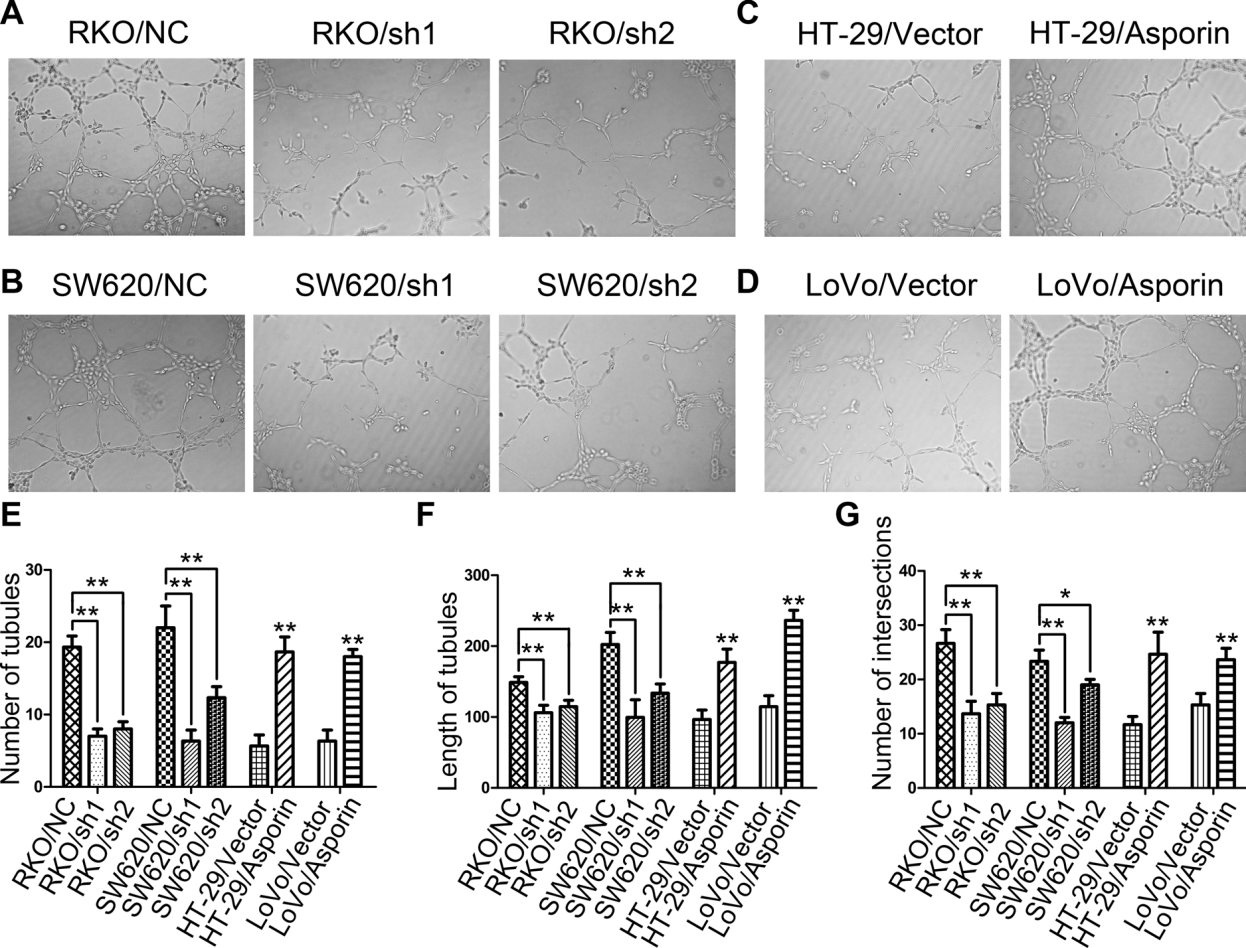
Asporin stimulates tubular formation *in vitro* (**A**–**D**) Representative photographs of tubular formation assays (200×). The numbers of tubules (**E**), mean tubular lengths (**F**) and intersecting nodes of tubes (**G**) between different groups. Data are represented as mean ± SD of three independent experiments. *p < 0.05; **p < 0.01.

To confirm whether asporin contributes to tumor angiogenesis, we performed tube-formation assays with human umbilical vein endothelial cells (HUVECs). After 8 hours incubation, the endothelial tube formation ability of HUVECs was quantified (Figure 4A, 4B, 4C and 4D). We found that the supernatant from HT-29/asporin, LoVo/asporin cells enhanced tubular formation of HUVECs compared with the control groups (Figure 4E, 4F and 4G). Opposite results were achieved in asporin knockdown groups (Figure 4E, 4F and 4G).

**Figure 5:**
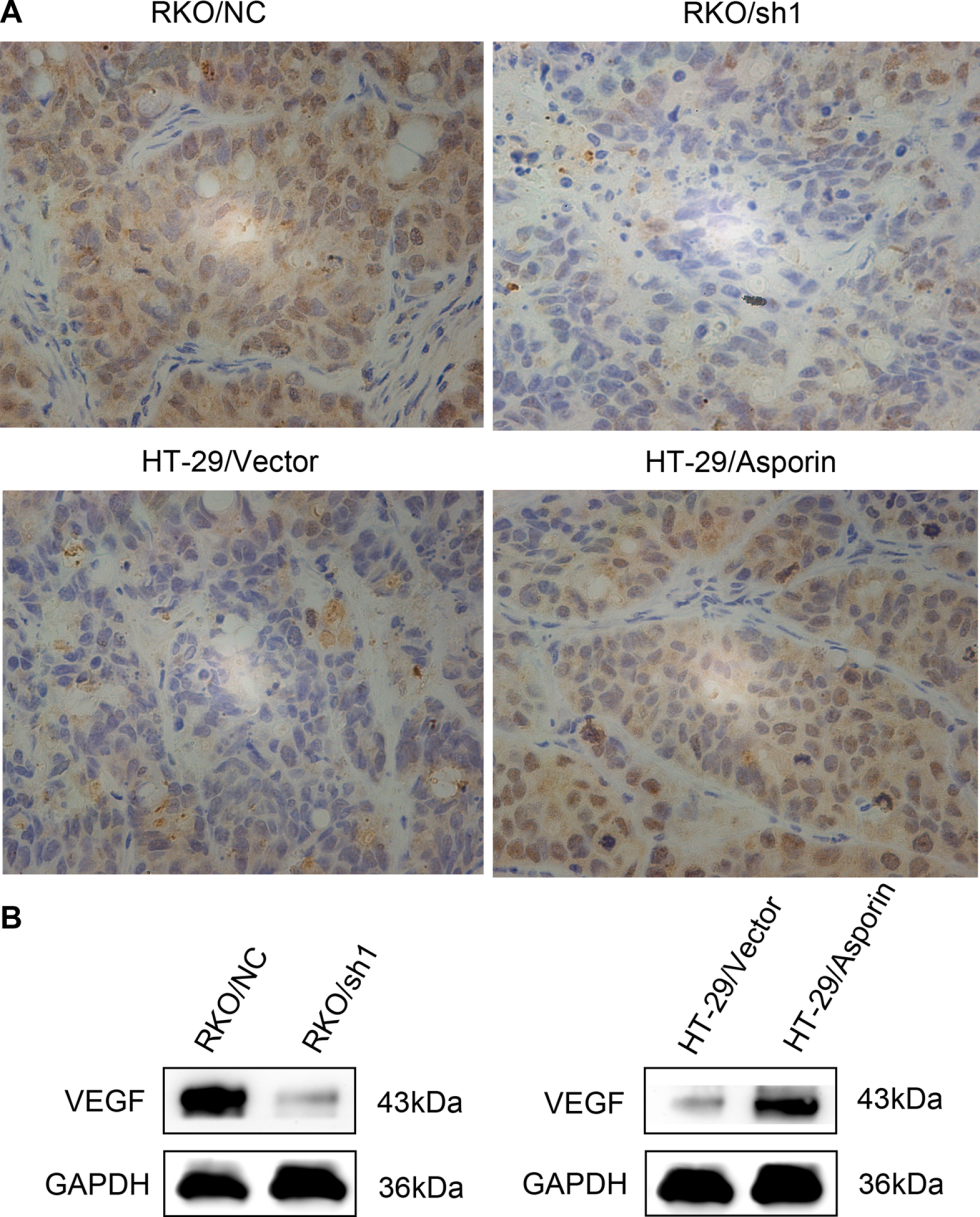
The expression of VEGF in subcutaneous tumors (**A**) The expression of VEGF in the subcutaneous tumor grafts was analyzed with immunohistochemical staining (400×). (**B**) Western blotting analysis of VEGF in the subcutaneous tumor grafts. GAPDH was used as a loading control.

We next tested whether asporin levels influence the angiogenesis ability of the CRC cells *in vivo*. We injected RKO/NC, RKO/sh1-asporin, HT-29/Vector, and HT-29/asporin cells subcutaneously into BALB/c nude mice. Both immunohistochemitry and western blotting results suggested that asporin activated VEGF expression in these subcutaneous tumors and promoted the tumor growth (Figure 5A and 5B).

### Asporin stimulates the cortactin signaling pathway

**Figure 6:**
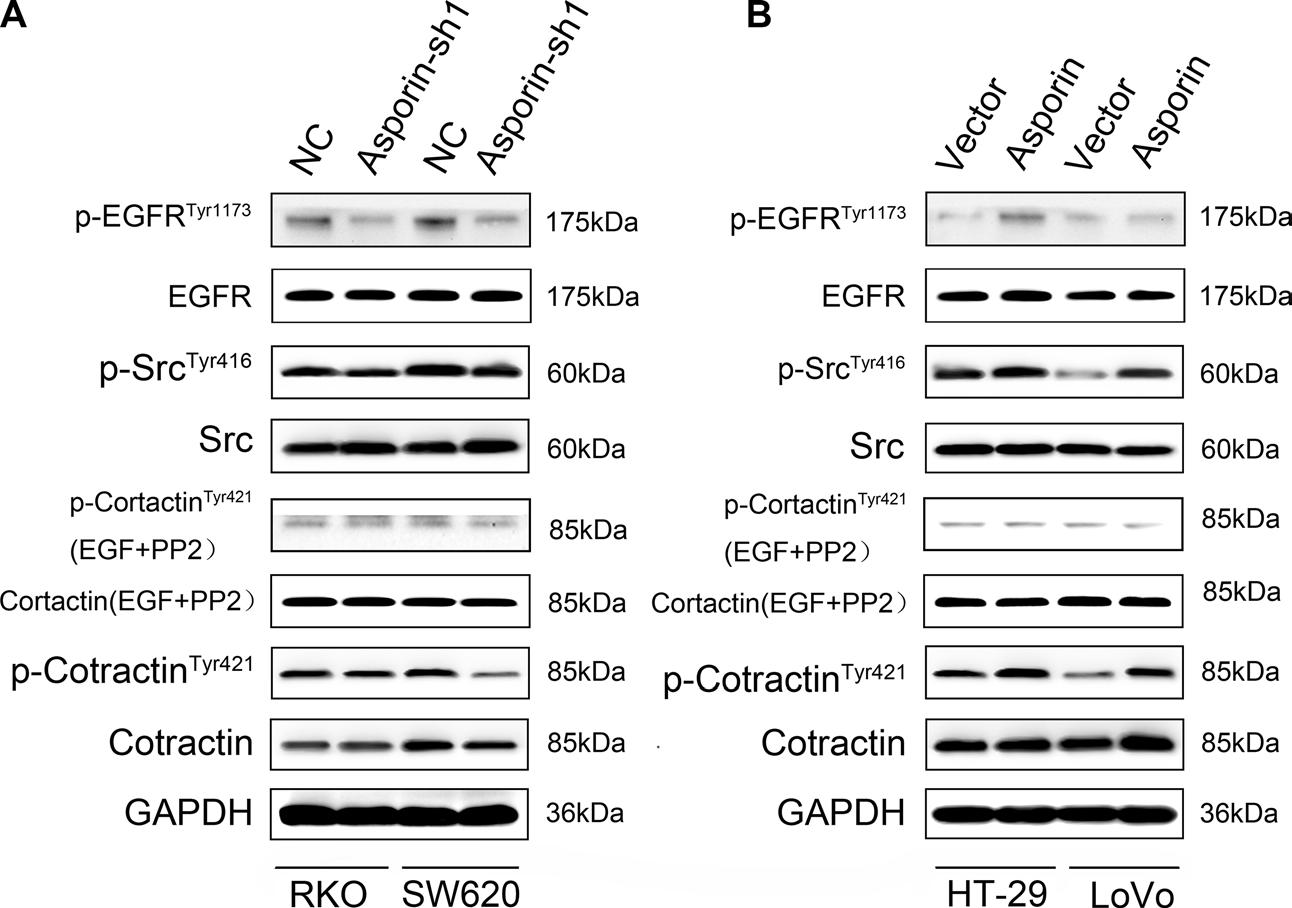
Asporin activates cortactin in CRC cells (**A**–**B**) Western blotting analysis of EGFR, phosphorylated EGFR, Src, phosphorylated Src, cortactin and phosphorylated cortactin in the cytosolic fraction. Serum-starved cells were stimulated with EGF (10 ng/ml) in the presence of PP2 (10 μM). GAPDH was used as a loading control.

We observed that the expression of phosphorylated (Tyr1173) EGFR, phosphorylated (Tyr416) Src significantly increased in HT-29/asporin, LoVo/asporin cells compared to control cells and they were decreased in asporin knockdown cells. Cortactin is a branched actin regulator associated with cell migration and invadopodia formation [14]. Augmentation of asporin expression increased the phosphorylation (Tyr421) of cortactin, but not the total cortactin level. The phosphorylation was abrogated in the presence of EGF and PP2, implying that EGFR/Src mediated asporin-induced cortactin activation (Figure 6A, 6B). These were consistent with the results that higher co-expression of asporin and p-cortactin (Tyr421) we identified in human CRC tissues (Figure 1F, 1G, 1H and 1I).

### Asporin stimulates the liver metastasis of CRC cells

**Figure 7:**
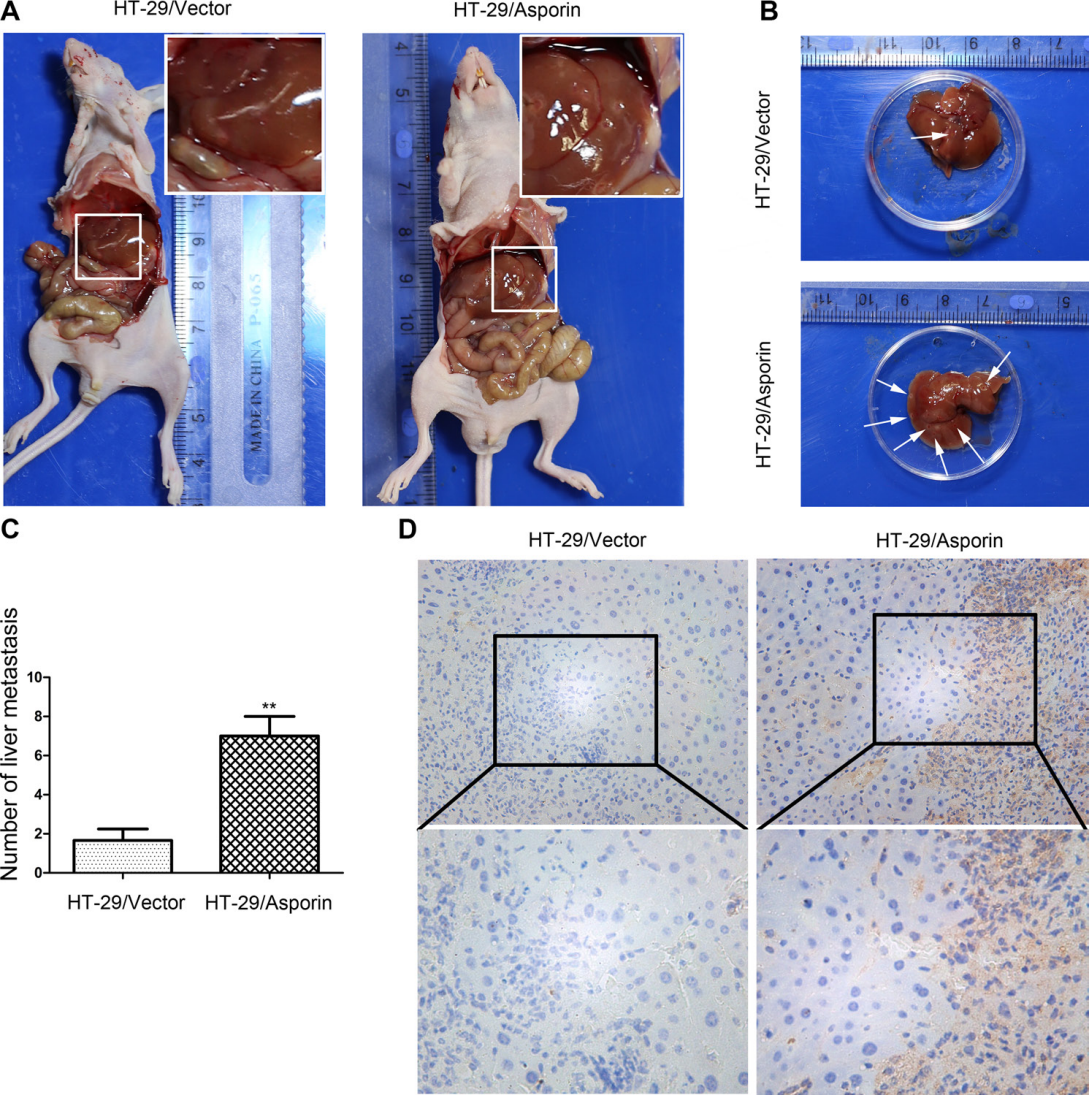
Asporin enhances the metastasis of colorectal cancer cell *in vivo* (**A**–**B**) Representative figures for observable liver metastases of CRC cells. (**C)** Statistical plot of observable liver metastases in each group. (**D**) The expression of asporin in metastatic tumors was quantified by immunohistochemical staining (200×). Data are represented as mean ± SD of three independent experiments. **p < 0.01.

As liver is one of the major metastasis organs of CRC cells, we applied the portal vein injection model to determine the functions of asporin in the liver metastasis of the CRC cells *in vivo*. Six weeks after injection, the mice were sacrificed and liver metastasis of the CRC cells was evaluated. As shown in Figure 7A, 7B, 7C and 7D, there was a significantly increment of liver metastatic nodules in mice injected with the HT-29/asporin cells compared to those injected with control cells. In contrast, knockdown of asporin could significantly decrease the number of liver metastatic nodules compared to the control cells (Supplementary Figure S3).

## DISCUSSION

Metastasis is an important biological characteristic of malignant tumors and responsible for as much as 90% of cancer-associated mortality [15]. SLRPs are biologically active components of ECM that are involved in the metastasis of multiple types of cancers [16]. In the present study, we found that asporin, one vital protein of SLRPs, was highly expressed in CRC tissues compared to the normal tissues. Clinical relevant studies suggested that asporin was significantly correlated with lymph node status and TNM stage of the patients. Gain- and loss-of asporin in CRC cell lines suggested that asporin promoted the cellular migration, invasion and metastasis. The *in vivo* xenograft experiments have suggested that asporin promoted the tumor growth through enhancing the production of VEGF levels, which was consistent with the results of the HUVEC tube formation assays. With the *in vivo* portal vein injection methods, we found that asporin promoted the liver metastasis of the CRC cells. To the best of our knowledge, this was the first study that had determined the roles of asporin in CRC development and progression.

The EGFR/Src signaling pathway was found to mediate the regulation of cortactin by asporin. Previously studies indicated that asporin might inhibit the TGF-β1 [17] and bone morphogenetic protein 2 (BMP-2) signaling pathways [8]. In gastric cancer, asporin contributes to metastasis through EGFR and ERK-CD44/MMP-2 pathways [9]. Awata et al. demonstrated that asporin could directly bind to FGF-2 and form an FGF-2–FGFR1 complex [18]. Phosphorylated EGFR recruited multiple Src homology 2 domains and activated their downstreaming signaling pathways [19]. Multiple studies had revealed that Src played crucial roles in the metastasis of many types of human cancers [20]. We found that asporin augmented phosphorylation of EGFR and Src in CRC cells. Given that TGF-β1 could reduce the phosphorylation level of cortactin [21], we hypothesized that cortactin might participate in the asporin-associated pathway. Cortactin exhibited essential roles in the formation of invadopodia [22], and enhanced the secretion of matrix metalloproteinases (MMPs) [23]. Cortactin is a substrate for Src, and phosphorylation of cortactin by Src occurs at three tyrosine residues, including tyrosine 421, 466, and 482 sites [24]. Interestingly, phosphorylation of tyrosine 421 and 466, the two primary phosphorylation residues, is a progressive process, while tyrosine 466 phosphorylation dependent on the phosphorylation of tyrosine 421 [24]. Here we found that asporin induced the phosphorylation of cortactin at tyrosine 421, which led to the activation of cortactin. EGFR activate Src, and it could be also activated by Src [25]. In the present study, the phosphorylation difference of cortactin was abrogated in the presence of EGF and PP2, the Src inhibitor. Our research demonstrated that asporin might promote cancer cell invasion by targeting the EGFR/Src/cortactin signaling pathway.

In conclusion, higher expression of asporin was noticed in CRC tissues and it was correlated with later clinical stage of the patients. Asporin promoted the migration and invasion of the tumor cells partially through an EGFR/Src/cortactin- signaling pathway. As a potential oncogene, asporin might be a prognostic biomarker for CRC patients and it was a potential therapeutic target for colorectal cancer treatment.

## MATERIALS AND METHODS

### Obtaining of tissue specimens

Tumor tissues and adjacent non-tumor tissues of 200 CRC patients were obtained from Ruijin Hospital, School of Medicine, Shanghai Jiaotong University. These patients had undergone radical surgery at Ruijin Hospital between January 2010 and January 2014, and were staged based on the TNM classification of the World Health Organization [26]. The mean age of the 200 patients was 63.5 years (rang: 30–81 years). None of the patients received neoadjuvant therapy such as radiation or chemotherapy before the surgery. This study was approved by the Ethics Committee of Ruijin Hospital, and informed consent was obtained from each CRC patient.

### Cell culture

The human CRC cell lines HT-29, LoVo, Caco2, HCT116, SW1116, SW480, RKO, SW620 and human umbilical vein endothelial cells (HUVEC) were obtained from American Type Culture Collection (Manassas, USA). These cell lines are preserved in the Shanghai Digestive Surgery Institute. HT-29 and HCT116 were cultured in McCoy's 5a Medium. LoVo and HUVEC were cultured in F-12K Medium. Caco2 and RKO were cultured in Eagle's Minimum Essential Medium. SW1116, SW480 and SW620 were cultured in Leibovitz's L-15 medium. All these media were supplemented with 10% fetal calf serum, 100 U/ml penicillin, 100 μg/ml streptomycin, and cultured at 37°C, with 5% CO_2_.

### Quantitative reverse transcription-polymerase chain reaction (qRT-PCR)

Total RNA was isolated from CRC tissues and cell lines using Trizol Reagent (Invitrogen, USA) according to the manufacturer's instructions. RNAs were then reverse transcribed into cDNAs using the reverse transcription kit (Promega, USA). Primers of asporin were 5′- CTCTGCCAAACCCTTCTTTAGC −3′ (forward) and 5′- CGTGAATAGCACTGACATCCAA −3′ (reverse). Primers of GAPDH were 5′- GGACCTGACCTGCCGTCTAG −3′ (forward) and 5′- GTAGCCCAGGATGCCCTTGA −3′ (reverse). GAPDH was used as an internal control. PCR reactions were carried out in the Applied Biosystems Prism 7500 Fast Sequence Detection System (Life Technologies Corporation, USA).

### Plasmids construction and viral infection

The short hairpin RNA (shRNA) and lentiviral transduction supernatant of asporin were obtained from GenePharma (Shanghai, China). Asporin sh1RNA (targeting sequence: GCTTACCACCAACTTTATTGG), sh2RNA (targeting sequence: GATGCTGAAG GATATGGAAGA) and negative control (targeting sequence: GTTCTCCGAACGTGTCACGT) were transduced separately into RKO and SW620 cells by Lipofectamine 2000 (Invitrogen, USA). Stable cell lines were respectively selected with 1 mg/mL G418 (Gibco, USA) and detected by Western blot. The asporin construct was generated by chemical synthesis, and was cloned into lentiviral vector and transfected into HT-29 and LoVo cells. Cells were selected with 4 μg/mL Puromycin (Acros, USA) and validated by Western blot.

### Immunohistochemistry

Tissues were fixed in 10% paraformaldehyde and embedded in paraffin after collection. The sections (5-μm) were then deparaffinized with xylene and rehydrated in alcohol. Blocking of endogenous peroxidase activity was performed with 3% H_2_O_2_ for 30 min. Antigen was retrieved by citrate buffer (pH = 6.0). The sections were incubated in the primary antibodies at 4°C overnight. The antibodies we used were as follows: anti-asporin (dilution 1:300, Abcam, USA), anti-p-cortactin (Tyr421, dilution 1:150, Merck Millipore, Germany). After incubation of biotinylated anti-rabbit secondary antibody, peroxidase activity was developed by DAB. Finally, slides were counterstained with Hematoxylin. Using the semi-quantitative immunoreactivity score (IRS), immunohistochemical staining was scored independently by two pathologists who had no knowledge of the patients' clinical data. Intensity of staining was evaluated as negative (0), weak (1), moderate (2) or strong (3). Percentage of positive cells was classified as follows: < 10% (0), 10–25% (1), > 25–50% (2), > 50–75% (3), and > 75% (4). By multiplication of two parameters, cases were grouped as negative (≤2), weak positive (2–5), and strong positive (> 5).

### Western blotting

Equal numbers (2.0 × 10^6^) of cells were lysed in RIPA (Sigma-Aldrich, USA) buffer containing 1% PMSF (Sigma-Aldrich, USA). Total protein concentration was quantified by BCA protein assay kit (Pierce, USA). Equal amounts (50 μg) of protein samples were injected into SDS-PAGE electrophoresis, and transferred onto polyvinylidene fluoride (PVDF) membranes. The membranes were blocked in 5% non-fat milk for 1 h, and incubated with primary antibodies overnight at 4°C. Primary antibodies we used were as follows: anti-asporin (dilution 1:1000, Abcam, USA), anti-VEGF (dilution 1:1000, Abcam, USA), anti-EGFR (dilution 1:1000, Cell Signaling Technology, USA), anti-p-EGFR (Tyr1173, dilution 1:1000, Cell Signaling Technology, USA), anti-Src (dilution 1:1000, Cell Signaling Technology, USA), anti-p-Src (Tyr416, dilution 1:1000, Cell Signaling Technology, USA), anti-cortactin (dilution 1:1000, Santa Cruz Biotechnology, USA), anti-p-cortactin (Tyr421, dilution 1:1000, Merck Millipore, Germany) and anti-GAPDH (dilution 1:10000, Abcam, USA). Membranes were then incubated in secondary antibodies at room temperature for 2 hours. Signals were visualized by an enhanced chemiluminescence detection system (Amersham Bioscience, USA) according to manufacturer's protocol.

### Wound healing assay

Cells were grown to confluence in 6-well plates. Three wounds were scratched for each sample using a sterile pipette tip. Cells were washed with PBS to remove the cellular debris and then cultured in serum-free medium. Migration of the cells was photographed at time-points of 0 hour, 24 hours and 48 hours.

### Migration and invasion assay

For migration assay, 2 × 10^4^ cells suspended in 200 μl serum-free medium were added into the upper transwell chamber (8 mm, Corning Costar, USA), and 500 μl medium supplemented with 10% serum was placed in the bottom chamber. After 24 h incubation, cells on the upper surface were removed with a cotton swab. Cells invading to the lower surface of membrane were fixed with methanol before staining with 0.4% crystal violet for 30 min. The stained cells were counted in 5 randomly selected fields under an inverted microscope. The invasion assay was performed as above except that cells were plated on the chamber precoated with Matrigel (BD Bioscience, USA).

### Endothelial tube formation assay

A thin layer of the Matrigel (50 μl/well, BD Bioscience, USA) was precoated in 96-well plates, and incubated at 37°C for 1 hour. HUVECs were resuspended in the supernant collected from each cell type. Equal amounts of supernant (300 μl) were added to each well at a concentration of 3 × 10^4^ cells/well. After incubation at 37°C with 5% CO_2_ for 8 hours, the number, length and intersecting nodes of tubes in 5 random fields were analyzed by Image Pro Plus software to evaluate tube formation.

### Xenograft model

Sixty male BALB/c nude mice at age of 4 weeks were purchased from the Institute of Zoology of the Chinese Academy of Sciences. Equal numbers of RKO/NC, RKO/sh1-asporin, HT-29/Vector, and HT-29/asporin cells (1 × 10^6^) were injected into the portal vein of ten BALB/c nude mice. Mice were sacrificed 6 weeks after injection. Livers were removed and metastatic nodules were analyzed. Equal numbers of RKO/NC, RKO/sh1-asporin, HT-29/Vector, and HT-29/asporin cells (2 × 10^6^) were injected subcutaneously into five BALB/c nude mice. Mice were sacrificed 4 weeks after injection. Subcutaneous tumor grafts were removed and analyzed by western blot and immunohistochemistry.

### Statistical analyses

The data were analyzed by SPSS 13.0 software. Differences between two groups were analyzed by Student's *t*-test. Chi-square or Fisher's exact tests was used to analyze correlation between asporin expression and clinical characteristics. Generally, *p* < 0.05 was considered statistically significant and *p* < 0.01 was considered highly significant.

## SUPPLEMENTARY MATERIALS FIGURES


