# Supplementary material for: Asporin enhances colorectal cancer metastasis through activating the EGFR/Src/cortactin signaling pathway
Source: Oncotarget. 2016 Sep 29;7(45):73402–13. doi: 10.18632/oncotarget.12336 (PMC5341987; doi:10.18632/oncotarget.12336)
Supplement: Supplementary file 1 [file oncotarget-07-73402-s001.pdf]

Asporin enhances colorectal cancer metastasis through activating the EGFR/Src/cortactin signaling pathway

Supplementary Materials

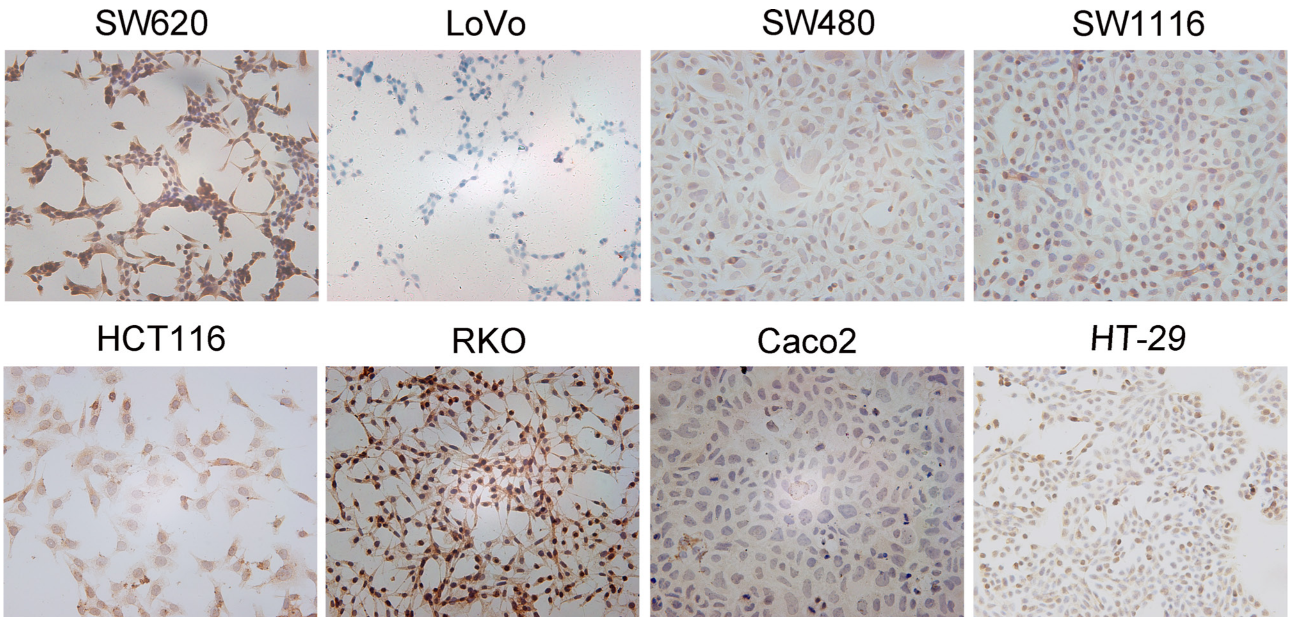

Supplementary Figure S1: Immunohistochemitry of eight colorectal cell lines.

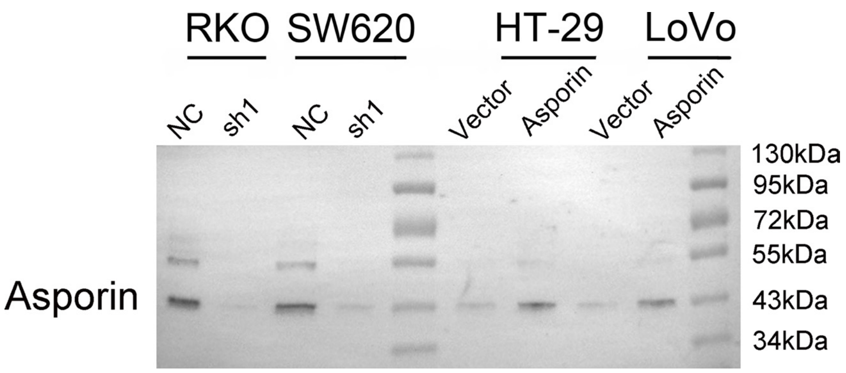

Supplementary Figure S2: Western blotting results of stable cells culture medium for asporin.

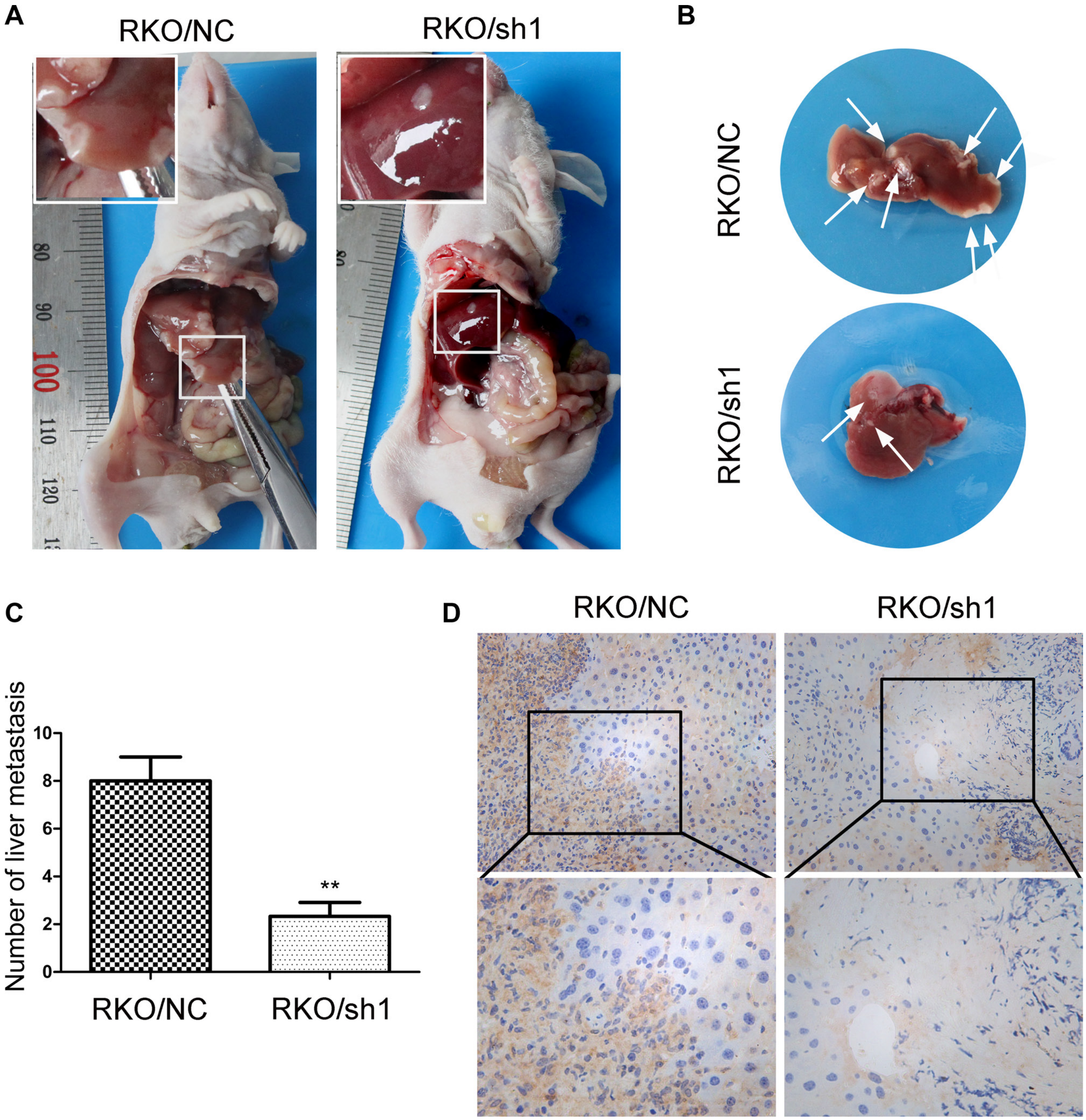

Supplementary Figure S3: Knockdown of asporin inhibits the metastasis of colorectal cancer cell *in vivo*. (**A**–**B**) Representative figures of observable liver metastases in each group. (**C**) Statistical plot of observable liver metastases in each group. (**D**) The expression of asporin in metastatic tumors was quantified by immunohistochemical staining (200×). Data are represented as mean ± SD of three independent experiments. $^{**}p < 0.01$.
